# Erythema multiform-like lesions in a patient infected with SARS-CoV-2: a case report

**DOI:** 10.2217/fvl-2020-0333

**Published:** 2021-03-09

**Authors:** Yaser Fathi, Elaheh Ghasemzadeh Hoseini, Reza Mottaghi

**Affiliations:** 1 ^1^Department of Oral & Maxillofacial Medicine, DDs, MSc, Assistant Professor, Alborz University of Medical Sciences, Tehran, Alborz, Iran; 2 ^2^Department of Oral & Maxillofacial Medicine, DDs, MSc, Assistant professor, School of Dentistry, Kashan University of Medical Science, Isfahan, Iran; 3 ^3^Department of Oral & Maxillofacial Surgery, DDs, Student Dentistry, Student Research Center, School of Dentistry, Kashan University of Medical Sciences, Isfahan, Iran

**Keywords:** atypical target, COVID-19, erythema multiform, hydroxychloroquine, hypersensitivity reaction, lip crusting, mucocutaneous lesion, oral ulceration, ribavirin, target lesion

## Abstract

**Background:** SARS-CoV-2, is followed by several manifestations, such as fever, cough, respiratory distress syndrome and mucocutaneous lesions such as papules, urticaria, vasculitic purpura and erythema multiform. **Case:** A 22-year old woman was diagnosed with COVID-19. Considering the skin and oral lesions, erythema multiform was suggested as the most likely diagnosis. Oral valaciclovir was administered. **Discussion:** Erythema multiforme were reported in some patients with COVID-19. Its pathophysiology is not yet completely understood, but it seems there is a lymphocyte-mediated hypersensitivity reaction to SARS-CoV-2 antigens presenting in the skin. **Conclusion:** Mucocutaneous and oral lesions might be the first manifestations of COVID-19. Therefore, during the pandemic, it is prudent to consider this virus as a differential diagnosis once we encounter oral ulceration.

## Lay abstract

We reported a 22-year old woman admitted to the hospital complaining of fever, abdominal pain, nausea and occasional vomiting that started about a week prior. According to the CT scan and real-time reverse transcription polymerase chain reaction (rRT-PCR), the patient was diagnosed with COVID-19. Oral examination by an oral medicine specialist revealed extensive ulcers in the mouth and crusts on the lips and she was diagnosed with erythema multiform. Oral valaciclovir was prescribed for 5 days and oral lesions relatively disappeared after 6 days. The pathophysiology of lesions is not yet completely understood, but it seems there is a hypersensitivity reaction to SARS-CoV-2 antigens presenting in the skin. SARS-CoV-2 could travel to other tissues such as skin, and make alterations to the cutaneous immune system, resulting in various manifestations on the skin.

In January 2020, the Chinese reported a new type of coronavirus that had not been detected in humans beforehand. The new virus, called SARS-CoV-2, as of 1 July 2020, has infected over 13 million people and resulted in about 500,000 deaths worldwide [1,2]. The patients with SARS-CoV-2 infection may present with various manifestations, for instance fever, flu-like symptoms, cough, respiratory distress syndrome, gastrointestinal symptoms, cardiovascular complications and mucocutaneous lesions [1]. Mucocutaneous manifestations of the disease, initially reported by Recalcati, are different, including papule, chicken pox, urticaria, vasculitic purpura, erythema multiform (EM) and reactivation of herpes virus [3,4].

EM is a hypersensitivity reaction that presents as an acute, self-limiting and sometimes recurrent condition. This delayed-type hypersensitivity reaction is triggered by certain infections (viral infection in particular), medications and some vaccines [5,6].

According to an international consensus classification, EM only is included major form and is characterized by severe mucosal involvement and its skin detachment is less than 10% of surface body (between 10 and 30% or more than 30% is defined toxic epidermal necrolyzis/Stevens Johnson syndrome overlap and Stevens Johnson syndrome/toxic epidermal necrolyzis respectively [7]. Undoubtedly, the new coronavirus, recently called ‘hit and run virus’, affects the immune system and may cause autoimmune damage to the host [8]. Considering the high mortality rate of COVID-19, early detection of skin and mucosal manifestations (which usually precede respiratory symptoms) could play an important role in early diagnosis and treatment of the disease [9].

## Case

**Figure 1.**
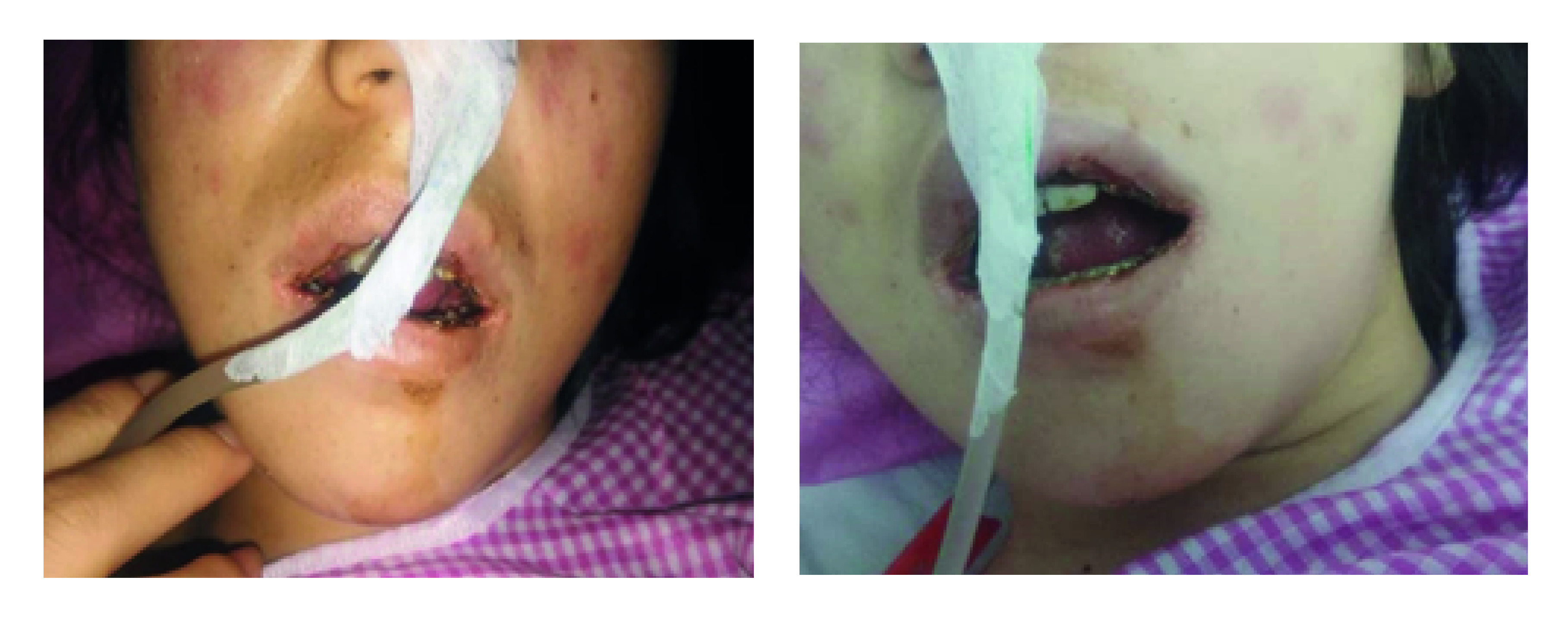
Hemorrhagic crusts on the lips. Erythematous lesions in the form of ‘atypical target’ were also observed on the facial skin.

**Figure 2.**
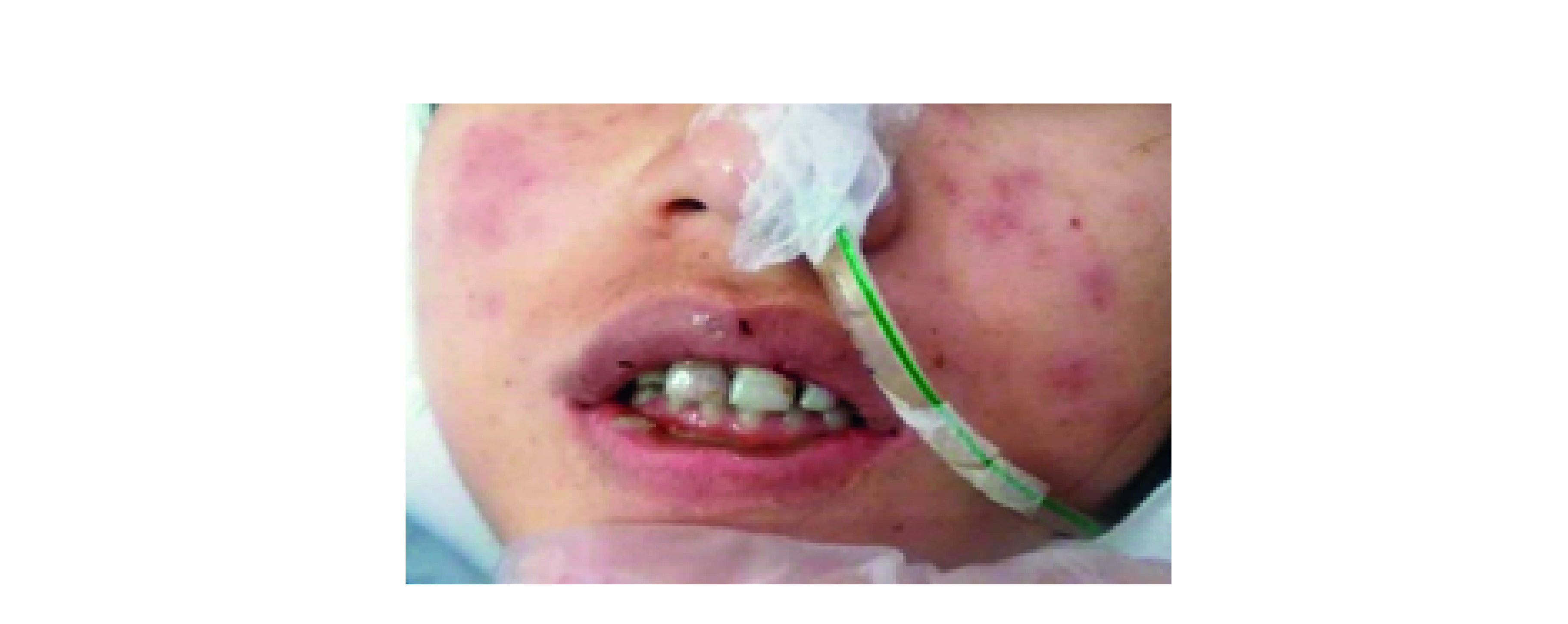
Erythematous lesions in the form of ‘atypical target’ on the facial skin.

The patient in this manuscript has given written informed consent to the publication of her case details and was reported after approval in ethical committee in Kashan university in 2020-08-11 (ID: IR.KAUMS.REC.1399.026). A 22-year old woman presented to the hospital in April 2020 complaining of fever, abdominal pain, nausea and occasional vomiting that had started about a week earlier. She had not taken any kinds of medications. Diagnosis of COVID-19 was established by RT-PCR in nasopharyngeal swab and lung CT scan. Complete blood cell count, coagulation and biochemical tests, hepatic and renal tests and abdominal ultrasound prescript for further evaluation of patient. Abdominal tenderness was observed during the examination. According to the test results and bilateral lung involvement on CT scan, the patient was diagnosed with COVID-19 and admitted in the ICU on the second day. Metronidazole, ceftriaxone, meropenem, ribavirin and hydroxychloroquine were administered and supplemental oxygen was given. On the third day, the patient complained of severe oral pain which was due to several ulcerative lesions developed in the oral mucosa. Oral medicine consultation was therefore carried out. Oral examination revealed extensive mucosal ulcers in the oral cavity and hemorrhagic crusts on the lips (Figure 1). Erythematous lesions in the form of ‘atypical target’ were also observed on the facial skin (Figure 2). Due to the possibility of infection transmission, biopsy was overlooked and the diagnosis was made based on the clinical features. In view of the skin lesions, oral ulcers and the hemorrhagic crusts on the lips, EM was suggested as the most likely diagnosis. The patient had no history of similar lesions or herpes simplex infection. According to her medical history, she had not used any medications or vaccines (in the past month). Considering the normal renal tests, oral valaciclovir was administered 500 mg twice a day. The removal of dental plaque and chlorhexidine mouthwash (twice a day) were also applied. Hepatic and renal tests (including ALT, AST, BUN, creatinine) were regularly monitored. Valacyclovir was continued for 5 days and oral lesions relatively disappeared after 4 days. However, the patient’s respiratory symptoms aggravated slightly while dyspnea and a mild unconsciousness appeared on the 10th day. Finally, following 32 days of hospitalization, the patient’s condition became stable and she was discharged from the hospital.

## Discussion

EM is a delayed-type hypersensitivity reaction commonly caused by certain infections (particularly herpes simplex and mycoplasma pneumoniae), medications (such as NSAIDs or antiepileptic drugs) and certain vaccines (such as the flu, mumps and measles) [5]. The disease generally affects adults aged between 20 and 40 years and involves other viral agents, such as VZV, CMV, HIV and hepatitis [5,6]. Following the COVID-19 pandemic, cases of EM were reported in patients with COVID-19, in recent months. This phenomenon was primarily described by Jimenez-Cauhe *et al.* as EM-like lesions in four patients with COVID-19. Subsequently, further similar cases were reported by other researchers, in which mucocutaneous lesions manifested before the typical symptoms of COVID-19 (fever and respiratory symptoms) [10]. These anonymous lesions, are commonly seen in children and young adults [11]. SARS-CoV-2, drugs such as hydroxychloroquine and oseltamivir and reactivation of HSV are some of the reasons found causing these lesions. Hydroxychloroquine is known as one of the drugs that cause EM [12]. Robutelli and colleagues reported EM in a patient with COVID-19 treated with hydroxychloroquine [13]. Patruno *et al.* suggested the possibility of better treatment outcomes for patients with COVID-19 who developed EM. According to this hypothesis, since eosinopenia plays a pivotal role in COVID-19 infection, drug-induced erythema multiform, which is associated with eosinophilia, can lead to better outcome of COVID-19. However, Jimenez and colleagues found no evidence to support this theory [14,15]. Demirbas *et al.* suggested the possible role of hydroxychloroquine and oseltamivir; however, they believed that SARS-CoV-2 is the leading cause of erythema multiform lesions [9]. Janah *et al.* described SARS-CoV-2 as a major cause of EM lesions even though they could not overlook the role of hydroxychloroquine [12].

The pathophysiology of the lesions is not yet completely understood, but there seems to be a lymphocyte-mediated hypersensitivity reaction to SARS-CoV-2 antigens presenting in the skin [12]. Immunohistochemistry (IHC) studies revealed SARS-CoV-2 spike protein in endothelial cells and epithelial cells of eccrine glands [16]. These findings are consistent with those of Gianotti’s histopathological study on cutaneous lesion specimens of COVID-19 patients. Gianotti *et al.* demonstrated that SARS-CoV-2 can travel to other tissues and organs through the vascular system. This migration may lead to destructive changes and make alterations to the cutaneous immune system. Hence, Langerhans cells of the skin become activated and cause various mucocutaneous manifestations [17].

## Conclusion

According to the recent studies, it could be concluded that mucocutaneous and oral lesions might be the initial manifestations of COVID-19, which present before other typical symptoms of the disease. Thus, during the COVID-19 pandemic, it is prudent to consider COVID-19 as a differential diagnosis in case of oral ulceration. The limitation of this study was the lack of serological tests for HSV and mycoplasma pneumonia (as we know mycoplasma induced rash may have similar clinical manifestations) and histopathological studies of mucocutaneous lesions which was due to the large number of COVID-19 medical emergencies. However, all these studies are primary steps to the identification of this new virus and further studies are required.

**Summary points**

- SARS-CoV-2 could travel to other tissues such as skin, and make some alterations to the cutaneous immune system resulting in various mucocutaneous manifestations.
- Mucocutaneous and oral lesions might be the initial manifestations of COVID-19.
- It is prudent to consider COVID-19 as a differential diagnosis in case of oral ulceration.
